# Bacteriophage Enumeration and Detection Methods

**DOI:** 10.3389/fmicb.2020.594868

**Published:** 2020-10-23

**Authors:** Norbert Ács, Michela Gambino, Lone Brøndsted

**Affiliations:** Department of Veterinary and Animal Sciences, University of Copenhagen, Copenhagen, Denmark

**Keywords:** bacteriophage, enumeration, detection, molecular biology, real-time PCR, sequencing, double agar overlay assay

## Abstract

Application of phages as alternative antimicrobials to combat pathogenic bacteria and their association to a healthy gut microbiome has prompted a need for precise methods for detection and enumeration of phage particles. There are many applicable methods, but care should be taken considering the measured object (infectious phage, whole phage particle or nucleic acid and proteins) and the concept behind the technique to avoid misinterpretations. While molecular methods cannot discriminate between viable and non-infectious phages, the traditional techniques for counting infectious phages can be time consuming and poorly reproducible. Here, we describe the methods currently used for phage detection and enumeration and highlight their advantages as well as their limitations. Finally, we provide insight on how to deal with complex samples, as well as future prospects in the field of phage quantification.

## Introduction

Bacteriophages (shortly phages, viruses that infect bacteria) are the most abundant biological entities in any habitat, reaching an overall abundance of 10^31^ viral particles (Hendrix et al., 1999; Suttle, 2007; Paez-Espino et al., 2016). While early studies of phage therapy performed in Eastern Europe (Stone, 2002) showed promising results against bacterial infections, their use has been largely overshadowed by antibiotics (Chanishvili, 2012). However, the overuse of antibiotics has led to the selection and spread of multidrug resistant bacteria and alternatives are urgently needed (Moellering, 2010). Therefore, phages are being re-discovered for medical applications, in cocktails or in combination with traditional antibiotics (Dickey and Perrot, 2019; Tagliaferri et al., 2019; Górski et al., 2020). In addition, there is a growing interest in manipulation of gut bacteria using phages, since the gut microbiome of humans and animals have been linked to a number of important factors such as neuronal development and several gastrointestinal diseases (Crohn’s disease, ulcerative colitis, irritable bowel syndrome) (Fujimura et al., 2010; Sharon et al., 2016; Zuo and Ng, 2018). To effectively assess the outcome of clinical treatments and to unravel the complex dynamics of microbial communities, detection and enumeration of phage particles in complex samples is of fundamental importance (Da̧browska, 2019). Furthermore, phage enumeration is needed for production and development of phage-based products, as well as for using phages for detection of bacterial infections and biocontrol of food products (Monk et al., 2010). Ideally, these methods should be based on reliable, robust and high-throughput procedures that effectively quantify phages, even in complex samples. Here, we describe the current methods for phage enumeration and detection (Table 1) and discuss the future prospects for new methods. We categorized the approaches based on the measured object: the infectious phage, the whole phage particle, the nucleic acid and proteins. In addition, we discuss the challenges that a complex sample pose to phage quantification. Most of these methods rely greatly on knowledge about the phage-host pair in advance. However, some molecular techniques are capable of investigating the whole virome in a given sample, or determining the viral count based upon genetic information only from the bacteriophage.

**TABLE 1:** Summary of enumeration methods discussed in the mini-review. For cost, an estimation is indicated, ranging from inexpensive ($) to highly expensive ($$$$).

Method	Basis of detection/enumeration	Duration	Manual labor	Cost	Advantages	Limitations	Reference for methodology
Double agar overlay assay (DLA)	Virulent phage particles	1–2 days	High	$	Simple, effective, “gold standard,” shows active virulence	Slow, laborious, high standardization needed for precise reproducibility	Kropinski et al., 2018
Transmission electron microscopy (TEM)	Magnification of virus particles	2–3 days	High	$$$	Works well with unknown phages	Costly, laborious, high concentration needed	Ackermann, 2012
Flow cytometry	Viral particles	4–12 h	Moderate	$$$	Can detect different phages in a sample	Expensive, low sensitivity, skilled operator needed	Brussaard et al., 2000
NanoSight	Nanoparticle detection by laser-illuminated optical microscopy	5–10 min	Low	$$$	Rapid runtime	Can be used only on clear, concentrated samples	Anderson et al., 2011
qPCR/RT-qPCR	Viral nucleic acid	2–6 h	Moderate	$$	Precise, reproducible	Overestimation of virulent particles (one magnitude)	Anderson et al., 2011
Droplet digital PCR (ddPCR)	Viral nucleic acid	2–6 h	Moderate	$$	No need for internal standards	Could easily overestimate viral abundance	Morella et al., 2018
Mass spectrometry	Viral protein	2–3 days	High	$$$	Accurate in determining PFU	Time-consuming, surface protein mutants can give false results	Wang et al., 2019
Illumina sequencing	Viral nucleic acid library	3–4 days	Moderate	$$	Not well suited for quantification	Significant amount of bioinformatics analysis needed	Klumpp et al., 2012
PacBio sequencing	Viral nucleic acid	2–5 days	Moderate	$$	Prone to sequencing errors	Long read lengths	Klumpp et al., 2012
NanoPore sequencing	Viral nucleic acid (can be amplified if needed)	8–24 h	Moderate	$$	Compact, rapid, multiple use	Expensive, high rate of sequencing read error	Ji et al., 2020

## Methods for Detection of Infectious Bacteriophages

Plaque counting is considered the golden standard for phage enumeration. The double agar overlay assay (DLA) allows localized phage-host contact in a confined environment (Petri dish) containing two layers of agar on top of each other. The bottom layer is prepared with medium supporting bacterial growth (for *Escherichia coli*, common media are lysogeny broth and brain heart infusion) containing 1–1.5% agar. The top layer contains the same medium with lower concentration of agar (0.4 to 0.6%), commonly referred to as soft-agar (Abedon and Yin, 2009) and it is mixed with the host bacterium and poured onto the bottom layer, resulting in a so-called lawn. In the top-agar, diffusion allows the bacteria to occupy the lawn completely and phages to bind to the bacteria. Samples containing phages are added on top of the second layer and dried or directly mixed with the bacteria and the soft agar. This is followed by incubation at the optimal temperature and time for bacterial growth (for *E. coli*, plates are incubated at 37°C overnight). If a phage is capable of infecting the tested bacterium, clear spots or plaques appear on the lawn, containing lysed bacteria and phage particles (Hyman and Abedon, 2009; Kropinski et al., 2018). A single plaque is the result of one phage initially lysing one host bacterium, and progeny phages killing neighboring cells subsequently. Larger clearing zones, instead, could be the result of other antimicrobial compounds in the sample or the presence of more phages mixed together. This is why it is fundamental to see, count and describe the single plaques. If the phage does not form clearly visible plaques, agar may be replaced by agarose, even in lower concentration (0.2%), as larger phages diffuse better in a softened environment (Serwer et al., 2007). Addition of divalent ions (CaCl_2_ and MgCl_2_) may also facilitate plaque formation through aiding phage adsorption to the bacterial receptor (Serwer et al., 2004). The concentration of infective phages (titer) in a phage stock or in a sample can be established by spotting serial dilutions of sample on a bacterial lawn. The concentration is expressed as plaque forming unit (PFU, analogous to the colony forming unit).

Enumeration of phages by the DLA method is a reference to develop novel methods. First, it allows to count the number of phages that can complete the whole lysis cycle in the tested conditions, from the binding to the synthesis and release of new virions infecting neighboring host cells. Then, it is simple yet efficient, it can be implemented in any laboratory with minimal investment costs and it is also applicable to many bacteriophages. On the other hand, it needs to be optimized to each phage-host pair, can be time consuming and may show high variability, if not well standardized. This is especially important when working with clinically relevant strains, for which the identification of infecting phages in a short period of time can be critical. To this aim, it should be taken into consideration that bacterial strains can mutate and develop resistance to the selected phages, thus making their application even more laborious if relies on a DLA method. On top of that, pipetting errors, operator bias, changes in bacterial growth parameters, contaminations from other bacteria and other phages can significantly impact the results (Anderson et al., 2011).

## Methods That Detect Whole Phage Particles

Transmission electron microscopy (TEM) uses a beam of electrons to produce 1000x higher resolution compared to traditional light microscopes. Increased resolution (down to 0.2 nm) is enough to visualize even viruses. This technology can be used to quantify viral particles, albeit the sample needs to be highly concentrated (∼10^6^ particles/mL) to produce reliable results (Mann, 2005; Goldsmith and Miller, 2009). Viral quantification using TEM is highly accurate in determining the morphotype and the total number, but it is considered time-consuming, expensive and impractical for running many samples and cannot be used for complex samples. Moreover, sample preparation is tedious and the technique requires a skilled operator on top of the sophisticated instrument (Ackermann, 2012).

Another technique to count whole particles is flow cytometry. Herein, viral particles are marked with a fluorescent dye and directed through a capillary. Small diameter of the capillary forces particles to flow in a single line, allowing detection of light scatter caused by each particle. The method is rapid and rigorous, thus employed widely (Picot et al., 2012). A seminal study showed that the fluorescent signal does not correlate with the genome size, but that different viruses in a mixed sample could be discriminated based on their fluorescence and side scatter distribution (Brussaard et al., 2000). Recently, it was shown that the fluorescent signal can be used to correlate the number of target nucleic acid molecules to the number of viral particles only if samples are handled gently, the use of surfactants is avoided, negative controls are included to determine auto-fluorescence of the medium and the instrument and assay sensitivity is estimated beforehand, using a panel of bacteriophages of various genome sizes (Dlusskaya et al., 2019).

Finally, a laser-based method developed by NanoSight Limited allows real time visualization and to enumerate viral particles in few minutes based on dynamic light scattering by laser-illuminated optical microscopy. Drawbacks are the need for relative high sample concentration (10^7^–10^9^ PFU/ml) and a clear liquid (Anderson et al., 2011) that is difficult to obtain from complex samples such as soil and fecal material.

## Methods Detecting Phage Nucleic Acid and Proteins

Polymerase chain reaction (PCR) is a simple and robust method to verify the presence of phages faster than plaque assays, based on the detection of nucleic acid. By applying random amplification of polymorphic DNA (RAPD) PCR, it is also possible to discriminate between different phage lineages in a matter of hours, with no need for whole genome sequencing (Gutiérrez et al., 2011). However, without a hallmark gene ubiquitously present in phages (like 16S rRNA gene in bacteria), it is cumbersome to design primers that target the whole phage diversity. Another important drawback of PCR is that results are not quantifiable, because the reaction has endpoint characteristics. However, with advancements in the field, quantitative PCR (qPCR) was developed. Analogously, the field of protein detection also progressed at a rapid pace. Liquid Chromatography with tandem mass spectrometry (LC-MS-MS) method can be used to precisely detect and enumerate peptides from an unknown sample.

### Quantitative PCR (qPCR)

The discovery of intercalating fluorescent dyes (such as SYBR green introduced in 1994) gave ability to measure the amount of DNA product polymerized after each PCR cycle (Jin et al., 1994). The fluorescence is recorded by appropriate thermocyclers, able to amplify nucleic acid and detect fluorescence in one step. Standards can be used as reference when calculating the initial DNA concentration (Mackay et al., 2002). There are two basic chemistries used throughout qPCR platforms. The first is based upon intercalating fluorescent DNA dyes, whereas the second operates with a probe, an additional short DNA fragment with a fluorescent dye and a quencher molecule attached to it. The use of intercalating dyes is less selective for quantification of double-stranded DNA, whereas using fluorescently labeled probes is more precise, since signal is only produced after successful hybridization of all three DNA fragments (forward primer, reverse primer and probe) and subsequent polymerization (Mackay et al., 2002). Both methods have been extensively used in phage biology. qPCR was selected to quantify bacteriophages M13 and T7 using both SYBR green and TaqMan chemistries on different genes of both phages (Peng et al., 2018). The authors reported higher number of phage particles compared to the PFU counts determined by DLA for both methods, with the SYBR green showing the highest. They suggested that the presence of non-infectious viral like particles might cause this discrepancy. DNase treatment on the intact phages was able to decrease the signal, but the difference was still relatively high (Peng et al., 2018). qPCR was also used to measure phage particles in human or mice serum containing anti-T4 antibodies and even from plastic surfaces treated with proteinase K (Kłopot et al., 2017). Since the phages were not infectious after being neutralized by the antibodies in the serum, they could not be quantified by DLA. This highlights the sensitivity of qPCR, but also its limits, not being able to distinguish between infective and defective viral particles.

Others tried to reduce the discrepancy between the DLA and qPCR by treating the sample for PCR with propidium monoazide (PMA). PMA can enter compromised bacterial cells, where it crosslinks DNA and prevents the polymerase from gaining access. Nevertheless, PMA treatment decreased the qPCR signal of T4 bacteriophage only after complete disruption of viral capsids with heat treatment at 110°C, thus showing that PMA alone cannot be used for discrimination between infective and defective phages (Fittipaldi et al., 2010). Others compared DLA, qPCR and laser-based nanoparticle assay (NanoSight – discussed in **section “Methods That Detect Whole Phage Particles”**) using three different phages infecting diverse bacterial hosts (Anderson et al., 2011). Surprisingly, the results depended on the phage investigated and their concentration. When compared to DLA, one phage was overrepresented, a second was underrepresented, while the third showed mixed results by qPCR, but contamination of the latter phage preparation or the PCR mixture could not be ruled out. Yet, the authors proposed a correction factor that could be applied to translate qPCR results to viable phage counts (Anderson et al., 2011). Finally, quantifying RNA phages using qPCR is also possible, as RNA can be reverse transcribed to cDNA and amplified in a single step, reducing hands-on time. Quantitative reverse transcription PCR (RT-qPCR) has been employed to detect and enumerate MS2 phage (Farkas et al., 2015).

### Droplet Digital PCR (ddPCR)

In droplet digital PCR (ddPCR), the sample is mixed with a hydrophobic substance to create water-oil emulsion and the reaction is carried out in each droplet simultaneously. The mixture is passed through a fluorescence detector to enumerate the number of droplets where amplification occurred over the total number of droplets. This is in proportion to the amount of template DNA in the sample, hence it can be used to calculate the initial concentration with no need for external standards (Kim et al., 2014). Using ddPCR to quantify the plant pathogen *Pseudomonas syringae* and two phages infecting this bacterium, a higher number (20 to 30 times higher) of phages is detected compared to DLA. The variation could be decreased to 3 to 4 times higher by DNase treatment before disrupting viral capsids, but it is still a considerable difference (Morella et al., 2018).

### Mass Spectrometry

Wang et al. (2019) proposed mass spectrometry to enumerate M13 phage via a short and a large peptide derived from the coat protein pVIII. Authors argued for the usefulness of this approach, however, the copy number of the protein needs to be determined before drawing conclusions regarding concentration. Mutants having different capsid structure could have distorting effect on results (Banu et al., 2014; Wang et al., 2019) and the method has not been applied so far on mixed samples.

### Next Generation Sequencing

Whole-genome sequencing (WGS) allows sequencing and assembly of complete phage genomes by bioinformatic analysis, without prior isolation. The Illumina sequencing technologies are frequently used to identify new phages and also to detect viruses from various habitats, but they are not optimized for enumeration. Yet, some information about abundance may be withdrawn from the number of assembled contigs, providing semi-quantitative data. Other limitations are due to loss of data during assembly of contigs, leading to underestimation of the number of phages in the sample. In addition, carryover of bacterial host DNA, lack of databases with reference phage genomes (Hatfull, 2008) and the mosaic nature of phage genomes makes viral WGS challenging as a method for enumeration and detection, unless the sequence is known beforehand (Dorscht et al., 2009). Recently, single molecule sequencing approaches (PacBio and Oxford Nanopore) with reads spanning the entire phage genome have become more broadly available (Klumpp et al., 2012). Oxford Nanopore offers compact, high-throughput machines and rapid runtime that may make this method the standard choice for both sequencing of purified single phage as well as phage enumeration and detection. MinION^TM^, a portable sequencer of Oxford Nanopore was used to detect RNA (MS2) and DNA (PhiX174) phages from water samples. Both phages were successfully detected even at low concentrations, 155 PFU/mL and 1 to 2 PFU/mL for MS2 and PhiX174, respectively. Authors explained the difference in detection threshold with disparity in recovery rate between the two bacteriophages, as RNA is more prone to nucleic acid degradation (Ji et al., 2020).

## Quantification in Complex Samples

For enumeration and detection of phages in complex samples, such as clinical specimens, feces or food, a specific treatment is needed due to the presence of chemical compounds that may impact the results (Wilson, 1997). In case of quantification of a single phage from a complex sample, it is a prerequisite to know the both the phage and its host to build a complete picture about their dynamics. If the interest, instead, is to quantify the total phage population, the main challenge is the removal of inhibitors. Usually, a centrifuge step removes larger particles and, when followed by filtration using polytetrafluoroethylene membrane filters (pore size 0.22 μm–0.45 μm), the liquid fraction is enriched with viral particles. It is common to concentrate the filtrate using polyethylene-glycol to obtain a dense viral suspension for their enumeration and detection using for example DLA (Conceição-Neto et al., 2015). Also for PCR based methods, physical separation should be applied before viral nucleic acid isolation to minimize the carryover of PCR inhibitors (Rådström et al., 2004). The enumeration of phages in complex samples using next generation sequencing can be affected by the storage conditions. When testing storage temperature, storage duration and multiple freeze-thaw cycles, only the time of storage had a modest, but statistically significant impact on the β-diversity of the microbiota in human fecal samples (Shkoporov et al., 2018). When applying next generation sequencing for enumeration of phages in complex samples, the bioinformatics workflow requires a pipeline that includes quality check, filter, assembly and identification of phage DNA sequences. An important step in the analysis is filtering of the reads to remove non-viral sequences, which may interfere with the subsequent analysis. This can be done by aligning against a 16S rRNA sequence database (Roux et al., 2013), but this is proven to be unreliable in many cases, especially when reverse transcription is carried out. Another bacterial household gene, *cpn60* was suggested to better estimate bacterial DNA contamination during bioinformatical analysis (Shkoporov et al., 2018). On top of this, sequences showing homology to human genome should also be excluded by filtration. To date, identification of phage sequences is based upon homology to existing databases, however, because of our limited knowledge of viral sequences, most of them cannot be assigned. Thus, quite often the majority of sequences are marked as “unknown” and omitted from the evaluation (Sutton and Hill, 2019), commonly referred to as viral dark matter (Krishnamurthy and Wang, 2017). To overcome this problem, Shkoporov et al. (2018) proposed an assembly-based approach that included their own contigs in the viral database if they met one or more criteria among the following: predicted as viral by Virsorter program, showing 50% identity over 90% of contig length to known sequences in the NCBI viral RefSeq database, being circular, having a 10 times coverage over 3 kb and generating no alignments longer than 100 nucleotides (with *e* value < 1e-10) to the NCBI nucleotide database. This approach helped to include almost 50% of all their Illumina reads into a curated viral database, thus significantly increasing the investigated amount of viral content in the sample. Selecting the proper pretreatment and downstream methods is important to obtain comprehensive results about phage concentration when dealing with complex samples. Following these guidelines, however, can help achieving comparable results throughout phage therapy studies.

## Concluding Remarks (Future Prospects)

There is a growing need for methods that allow phage enumeration and detection in complex samples, due to recent development in clinical use of phages for treating bacterial infections as well as use of phages for biocontrol in food. The most widely used choice is the DLA method, due to its advantages of being fast, inexpensive and accurate in determining virulent phage counts. In parallel, several molecular biological techniques have improved speed and reproducibility. However, it is a disadvantage that these methods cannot discriminate between infective and defective virus particles, hence giving results that are hard to compare to traditional DLA plaque assay. Improvements in molecular and phage biology will help to develop new methods that combine advantages of currently used enumeration and detection techniques. Until then, if a rapid high throughput assay is needed to enumerate a specific phage in multiple samples, we suggest using qPCR with the addition of a DLA confirmed correction coefficient, to translate the results to viable phage counts.

## Author Contributions

NÁ and MG performed the necessary literature searches and conceptualized and wrote the first draft of the manuscript. LB critically revised the manuscript and made significant improvements. All authors read and approved the submitted version of the manuscript.

## Conflict of Interest

The authors declare that the research was conducted in the absence of any commercial or financial relationships that could be construed as a potential conflict of interest.
